# Heavy metals and natural radioactive isotopes in phosphate and compound fertilisers and their impact on human health

**DOI:** 10.1038/s41598-025-18945-4

**Published:** 2025-09-29

**Authors:** Aneta Łukaszek-Chmielewska, Marzena Rachwał, Joanna Rakowska, Robert Piec, Anna Podleśna, Barbara Piotrowska, Krzysztof Isajenko, Jerzy W. Mietelski, Renata Kierepko, Bogdan Kosturkiewicz

**Affiliations:** 1 Fire University, 52/54 Słowackiego St., Warsaw, 01-629 Poland; 2 Institute of Soil Science and Plant Cultivation, State Research Institute, 8 Czartoryskich St., Puławy, 24-100 Poland; 3 Central Laboratory for Radiological Protection, 7 Konwaliowa St., Warsaw, 03-194 Poland; 4 The Henryk Niewodniczanski Institute of Nuclear Physics, Polish Academy of Sciences, 152 Radzikowskiego St., Cracow, 31-342 Poland; 5 AGH University of Kraków, 30 Al. Mickiewicza, Kraków, 30-059 Poland

**Keywords:** Fertilisers, Heavy metals, Natural radioactivity, Hazard indicators, Safety engineering, Chemistry, Environmental sciences

## Abstract

The paper presents the results of heavy metals (Ni, Cr, Pb, Cd, Zn and Cu) and natural radioactive isotopes (^238^U, ^226^Ra, ^232^Th and ^40^K) in phosphate and compound fertilisers produced in Poland. Heavy metals in the mineral fertilisers were determined by atomic absorption spectrophotometry in the acetylene-air flame. The content of natural radioactive isotopes was determined using gamma-ray and alpha spectrometry techniques. The average heavy metal content of the tested fertilisers was 154 ± 17, 154 ± 30, 5.43 ± 0.43, 7.87 ± 1.95, 197 ± 19 and 26 ± 7 mg$\cdot$kg^− 1^ for Ni, Cr, Pb, Cd, Zn and Cu, respectively. Whereas, the average concentrations of natural radioactive isotopes in the studied samples for ^238^U, ^226^Ra and ^40^K were respectively in the range of 715 ± 259, 549 ± 170 and 2472 ± 759 Bq$\cdot$kg^− 1^, and were higher than their average content in the Earth’s crust, in contrast to the average concentration of the 232Th isotope (12 ± 2 Bq$\cdot$kg^− 1^) in the tested samples compared to the average concentration in the Earth’s crust. To assess the health exposure of people in direct contact with fertilisers (factory workers, people working in fertiliser loading and unloading, transporters, storekeepers or farmers), basic radiological protection parameters such as radium equivalent activity (Ra_eq_), outdoor dose rate (D_out_), indoor dose rate (D_in_), annual outdoor effective dose and annual indoors effective dose (E_out_ and E_in_) and total annual effective dose (E_tot_) were determined. It was observed that the calculated radiological hazard parameters for all samples exceeded the worldwide average values.

**Supplementary Information:**

The online version contains supplementary material available at 10.1038/s41598-025-18945-4.

## Introduction

Fertilisation is a basic agronomic treatment and fertiliser use is one of the parameters for assessing farming intensity. According to data presented in the UN Department of Economic and Social Affairs’ World Population Prospects report, the human population is projected to grow soon, reaching 8.6 billion in 2030 and 9.8 billion in 2050^1^. This poses a huge challenge in terms of meeting the food needs of such a huge population. To meet the challenge, it is essential to maximise the production potential of plants. This can be achieved, among other things, through the rational use of mineral fertilisers. The consumption of NPK-type mineral fertilisers varies geographically. An increase in the level of fertilisation is currently being noticed in areas where it has been relatively low until now. These are primarily East Asia (31%), South Asia (20%), Latin America and the Caribbean (14%)^2^. Poland’s share in world production of mineral fertilisers was 1.4% in 2021, including nitrogen fertilisers – 1.8%^3^. Poland is ranked among countries with average mineral fertiliser consumption. However, Poland’s share of the world’s total fertiliser consumption is small, amounting to 0.9%, and in comparison with EU countries, it is 12.3%^4–6^. Data published by the Institute of Soil Science and Plant Cultivation - State Research Institute in Puławy^7^ indicate that in the 2021/2022 season, the consumption of mineral fertilisers in Poland decreased by approximately 11% compared to the 2020/2021 season, reaching 1.74 million tonnes in 2022/2023, mineral fertiliser consumption decreased by a further 8% and amounted to approximately 1.6 million tonnes.

Mineral fertilisation, besides the positive effects associated with improved soil fertility, can harm the environment and human health. Phosphate and compound fertilisers may contain elevated heavy metal contents (especially cadmium)^8,9^ and elevated concentrations of the natural radioactive isotopes radium (^226^Ra), thorium (^232^Th) and potassium ^40^K), which may result in an increased natural background of ionising radiation in the environment. The elevated natural radioactivity and heavy metal content in mineral fertilisers, especially cadmium in phosphate fertilisers, depend on the raw materials used in their production. These materials include the type of phosphate rock used, such as apatite and phosphate rock. Notably, apatites are minerals of volcanic origin, whereas phosphate rocks are minerals of sedimentary origin. Sedimentary phosphate ores usually tend to have high concentrations of ^238^U, whereas magmatic ores such as apatite do not. Typical activity concentrations of ^238^U are 1500 Bq kg^− 1^ in sedimentary phosphate deposits and 70 Bq kg^− 1^ in apatite. ^232^Th and^40^K in sedimentary phosphate rocks are much lower than those of ^238^U, and comparable to those typically observed in soil^10^. Studies of the natural radioactivity of phosphate rocks, as well as of phosphate and/or compound mineral fertilisers themselves, are very popular worldwide, as evidenced by the abundance of literature^11–32^. Whereas, in Poland, the issue of natural radioactivity of mineral fertilisers has not aroused high interest among scientists so far, as evidenced by the small number of papers on this subject^22,30^.

This study aims to assess the concentrations of natural radioactive isotopes of uranium (^238^U) radium (^226^Ra), thorium (^232^Th) and potassium ^40^K) and the content of heavy metals (Ni, Cr, Pb, Cd, Zn and Cu) in phosphate (SSP, TSP) and compound fertilisers (NPK) produced in Poland. Based on the determined concentrations of natural radioactive isotopes in the studied fertilisers, the authors calculated/determined basic parameters of radiological protection, such as radium equivalent activity (Ra_eq_), outdoor dose rate (D_out_), indoor dose rate (D_in_), annual outdoor effective dose, annual indoor effective dose (E_out_ and E_in_) and total annual effective dose (E_tot_). These parameters can be used to assess the degree of human exposure to ionising radiation emitted by the studied materials. It should be noted that during handling, packing and transporting of fertilisers, some workers can receive additional external exposure to radiation. Therefore, it is important to measure natural radioactivity in fertilisers, because the high radioactive content may lead to significant exposure of miners, manufacturers and end users. Furthermore, such research provides basic data for the estimation of the amount of radioactivity spread on agricultural land with fertilisers. The concentrations of natural radioactive isotopes of uranium, radium, thorium and potassium, as well as the concentration of heavy metals contained in fertilisers, were then compared with the results of studies by other authors and with legislative regulations in force in Poland and worldwide.

## Materials and methods

### Sample collection and preparation

Nine samples of commercially available Polish chemical fertilisers were collected from local markets. The investigated sample types are single super phosphate (SSP), triple super phosphate (TSP) and fertilisers containing different amounts of nitrogen, phosphorus and potassium (NPK).

Samples were prepared in different ways depending on the requirements of the measurement techniques used. However, the first step was to grind and sieve the samples.

For gamma-ray spectroscopy, the fertiliser samples were ground to a fine powder by using a grain mill and sieved through a sieve with a mesh diameter of 200 μm. Measurements of the activity concentration of natural radionuclides radium (^226^Ra), thorium (^232^Th) and potassium ^40^K) in the studied samples were carried out in closed, polypropylene containers in the shape of a truncated cone with a volume of 125 cm^3^. Each time, depending on the density of the tested material, about 124 to 167.5 g of the sample was used for measurement.

Whereas, for alpha spectroscopy, all fertiliser samples were ashed at 600˚C for 4 h. The mass losses due to ignitions were determined.

### Stable elements determination method

Nitrogen, phosphorus, and potassium content were determined in accordance with applicable procedures. Total ammonium nitrogen (N) was determined by titration according to standard EN 15475:2009. Phosphorus in the oxide form (P_2_O_5_) was determined gravimetrically in a neutral solution of ammonium citrate and water according to EN 15959:2011. Potassium in the oxide form (K_2_O) was determined according to EN 15477:2009. Whereas, heavy metals, i.e. Pb, Cd, Cu, Zn, Ni, and Cr, were determined by the ASA method after prior mineralisation of the samples in a mixture (1:1) of nitric (V) HNO_3_ and chloric (VII) HClO_4_ acids according to ISO 11466:1995 and soluble forms of the same heavy metals after extraction with 1 M HCl solution and ISO 11047:1998.

### Determination of the natural radionuclide concentrations

The analyses were conducted in accredited laboratories (accreditation No. AB 979 and AB 1215 issued by the Polish Centre of Accreditation) using gamma and alpha spectrometry. For studies not falling within the scope of the accreditation certificate, the laboratory employs non-accredited methods which comply with the applicable standards set out in the PN-EN ISO/IEC 17025:2018-02. In order to ensure the necessary traceability of measurements, a series of intercomparison measurements were performed through participation in the proficiency test conducted by the International Atomic Energy Agency (i.e. IAEA-TERC-2024-01, IAEA-TERC-2023, IAEA-RER7014-TC-TEL-2020-05). Since 2014, the laboratory has been authorised to use the ILAC (International Laboratory Accreditation Cooperation) mark. To verify the accuracy of the measurements, a range of reference materials were utilized (i.e. IAEA-434).

#### The gamma-rays measuring

Measurements of the activity concentration of natural radionuclides of radium (^226^Ra), thorium (^232^Th) and potassium ^40^K) in the studied samples were carried out in closed, polypropylene containers in the shape of a truncated cone with a volume of 125 cm^3^. Depending on the density of the material being tested, the sample weight ranged from approximately 124 to 167.5 g. The time of each measurement was 80 000 s. To determine the activity concentration of natural radionuclides (^226^Ra, ^232^Th and ^40^K) in the tested fertiliser samples, the gamma-ray spectrometry technique was used with two spectrometers equipped with semiconductor germanium detectors (HPGe type XTRa) with relative efficiencies of 30 and 40%. Energy calibration was performed using a certified source containing a mixture of gamma radioisotopes with emission energies covering the range 60–1836 keV. Due to the lack of a radioactive standard source with geometry appropriate to the described application, it was decided not to perform efficiency calibration using a standard source with a specific geometry. Instead, the measurement geometry was simulated in Canberra’s Laboratory Sourceless Calibration Software (LabSOCS). The LabSOCS program is used when it is impossible to provide a measurement geometry that would fully correspond to the measured samples. This software is able to mathematically simulate (create) a performance curve for a given type of sample and a given measurement geometry. Previous research proves that the LabSOCS software is a very good replacement for classic efficiency calibration using radioactive sources with specific geometry, and the results obtained using both methodologies are characterised by low discrepancies (4–10%)^33–35^. The use of LabSOCS software, instead of the standard calibration using radioactive sources, is an additional time-saver for sample testing. This almost immediate ability to generate new performance calibrations can contribute to faster measurement results for unusual samples. Instead of the long process of a standard performance calibration (which includes measuring the source, if applicable, creating performance curves, etc.), a LabSOCS calibration requires minimal time expenditure in front of a computer. The utilisation of this tool facilitates the determination of the optimal sample container size, thereby maximising performance and simultaneously reducing measurement time.

The measurement uncertainties in gamma radiation measurements were expanded uncertainties (k = 2).

#### The alpha spectrometric measurements

To determine ^238^U concentration, about 2 g of each ashen sample was taken and subjected to a labour-intensive radiochemical procedure. Its application is due to the small penetration range of alpha particles and consists of several basic steps, such as mineralisation of fertiliser samples, sequential separation and purification of radionuclide, and application of uranium onto stainless steel discs. Afterwards, ^238^U concentration was measured using Ortec 576 alpha spectrometer equipped with silica PIPS detectors.

The uncertainty of alphaspectrometric measurements includes two distinct interconnected domains: measurement statistics and the uncertainty of the marker’s activity. For a considerable period, gravimetric control of the pipetted marker mass (with precision down to a single per mille) has been used, so the uncertainty of the marker’s activity is dominated by a factor of approximately 1% resulting from the manufacturer’s certificate (dilutions are also executed using gravimetric control).

### Calculation of radiological hazard parameters

In order to assess human exposure to ionising radiation from natural radioactive isotopes ^226^Ra, ^232^Th and ^40^K contained in the tested fertiliser samples, the basic parameters of radiological hazard were determined. These indices were radium equivalent activity (Ra_eq_), outdoor dose rate (D_out_), indoor dose rate (D_in_), annual outdoor and annual indoor effective doses (E_out_ and E_in_), and total annual effective dose (E_tot_). Table 1 presents the formulas used to calculate the radiological protection parameters. The following determinations were introduced: A_(Ra)_ - radium isotope concentration (^226^Ra), A_Th_ - thorium concentration (^232^Th) and A_K_ - potassium isotope concentration ^40^K) in the tested fertiliser samples.

**Table 1:** Formulas for calculating radiological protection factors.

Indicator/parameter	Formula	Numbering	Bibliography
Radium equivalent activity	$\:{Ra}_{eq}={A}_{Ra}+{1.43A}_{Th}+{0.077A}_{K}$	(1)	^36^
Outdoor external dose	$\:{D}_{out}=0.462{A}_{Ra}+0.604{A}_{Th}+0.0417{A}_{K}$	(2)	^37^
Indoor external dose	$\:{D}_{in}=0.92{A}_{Ra}+1.1{A}_{Th}+0.080{A}_{K}$	(3)	^38^
Annual effective dose equivalent outdoors	$\:{E}_{out}={D}_{out}\cdot\:8766\cdot\:0.8\cdot\:0.7\cdot\:{10}^{-6}$	(4)	^37^
Annual effective dose equivalent indoors	$\:{E}_{in}={D}_{in}\cdot\:8766\cdot\:0.2\cdot\:0.7\cdot\:{10}^{-6}$	(5)	^37^
Total annual effective dose equivalent	$\:{E}_{tot}={E}_{out}+{E}_{in}$	(6)	^37^

A more comprehensive exploration of radiological protection indicators can be found in the preceding studies^39–41^.

## Results and discussion

All measurement results and calculation data are summarised in the supplementary table.

## Content of stable elements

### Nutrients

Table 2 summarises the types of fertilisers tested and the content of the basic nutrients included in their composition, i.e. total nitrogen, phosphorus designated as P_2_O_5_ and potassium as K_2_O. SSP and TSP fertilisers are sources of phosphorus, with TSP fertilisers containing twice as much phosphorus (39.28–40.32%) as SSP fertilisers (17.72–18.68%). NPK fertilisers contain much less phosphorus, but are a rich source of nitrogen (3.93–11.84%) and potassium (13.60–20.64%).

**Table 2:** The types of fertilisers studied and their composition (nutrients).

Sample code	Fertilizer type^*^	Composition of fertilizer (%)		
		*N*	*P*_2_O_5_	K_2_O
F1	SSP	-	17.72 ± 1.42	-
F2	SSP	-	18.68 ± 2.24	-
F3	TSP	-	40.32 ± 3.22	-
F4	TSP		39.28 ± 3.14	-
F5	NPK	4.29 ± 0.30	8.88 ± 0.71	19.32 ± 1.16
F6	NPK	4.40 ± 0.31	9.94 ± 0.80	13.60 ± 0.82
F7	NPK	3.93 ± 0.28	9.00 ± 0.72	20.34 ± 1.22
F8	NPK	4.64 ± 0.32	10.10 ± 0.81	20.54 ± 1.23
F9	NPK	11.84 ± 0.83	10.32 ± 0.82	17.56 ± 1.05

*SSP - Single Superphosphate, TSP - Triple Superphosphate, NPK - Nitrogen, Phosphorus and Potassium.

#### Heavy metals

**Fig. 1:**
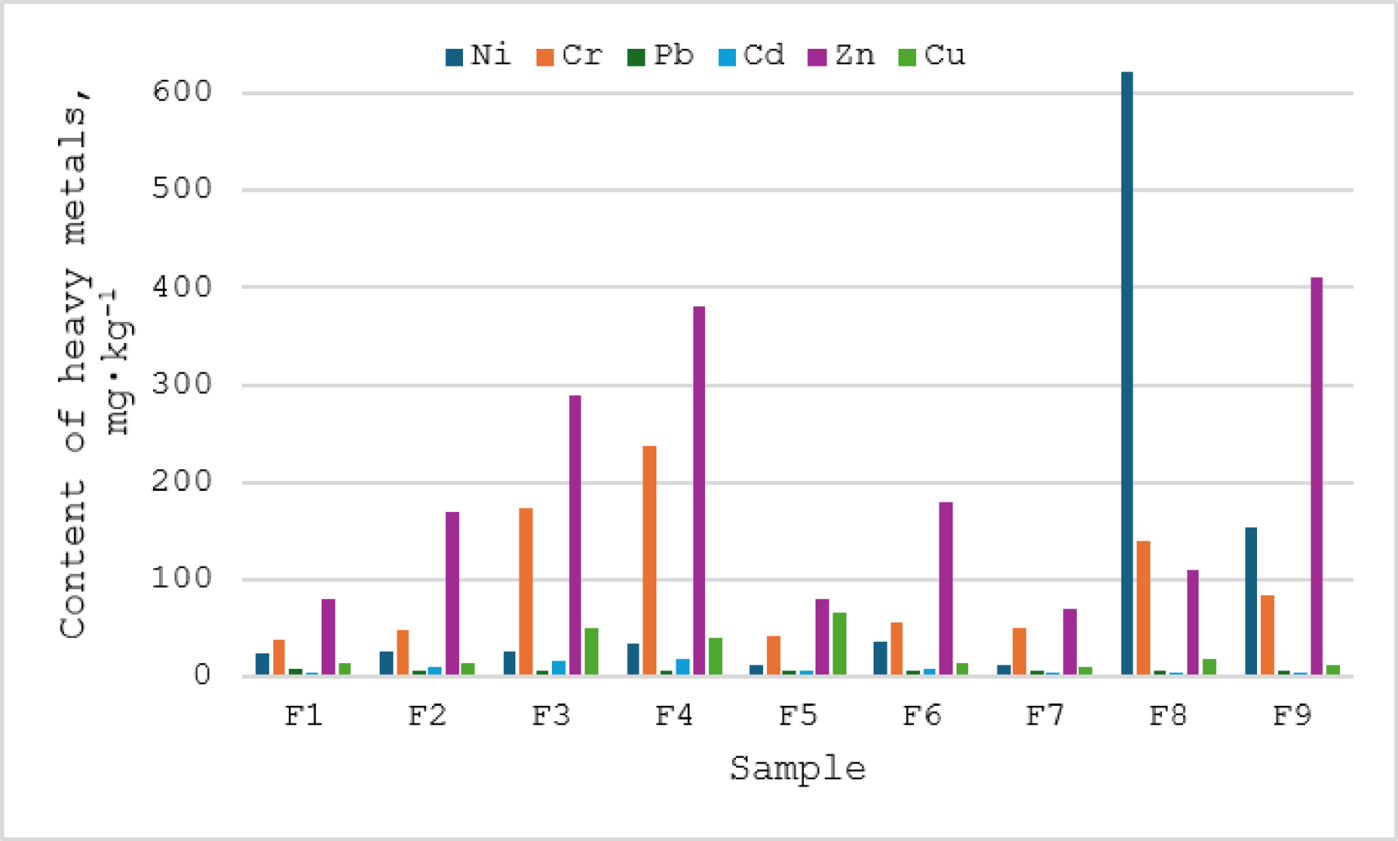
Heavy metal content in mineral/synthetic fertilisers.

According to literature data^9,42^ among mineral fertilisers, phosphate and compound fertilisers, which are the source of phosphorus, tend to contain more heavy metals, especially cadmium in the form of various impurities. Heavy metals contained in fertilisers originate from the raw material used in their production, but their additional source may also be the technological process. The production of phosphate fertilisers involves the utilisation of phosphorites, which, depending on the mining location, contain 5–80 mg of Cd in 1 kg^9^. Another potential source of heavy metal contamination of fertilisers may be the technological process during which metal cations, e.g. nickel and chromium, are released from the surface of the apparatus. The studied fertilisers were characterised by different contents of selected heavy metals i.e. nickel, chromium, lead, cadmium, zinc and copper (Fig. 1).

The phosphate and compound fertilisers tested contained on average the highest levels of zinc, nickel and chromium, and much lower levels of copper, cadmium and lead. Limits for the occurrence of contaminants in mineral fertilisers in Poland are determined by Regulation of the European Parliament and of the Council^43^. Inorganic macronutrient fertilisers may contain nickel up to 100 mg kg^− 1^, lead up to 120 mg∙kg^− 1^ and mercury less than 1 mg∙kg^− 1^ of fertiliser. The permitted content of chromium Cr (VI) is 2 mg∙kg^− 1^ and arsenic 40 mg∙kg^− 1^. If the total inorganic phosphorus content of the macronutrient fertiliser, calculated as P_2_O_5_, is less than 5% then the allowable cadmium content is 30 mg∙kg^− 1^ dry weight, and if this value is greater than or equal to 5% then the allowable cadmium content is 60 mg∙kg^− 1^ dry weight of the fertiliser^43^. Polish Regulation of the Minister of Agriculture and Rural Development^44^ determined accepted content of heavy metals in mineral fertilisers as 50 mg∙kg^− 1^ arsenium (As), 50 mg kg^− 1^ cadmium (Cd), 140 mg∙kg^− 1^ lead (Pb), and 2 mg kg^− 1^ mercury (Hg).

The results of nickel determinations in the tested samples vary and range from 11.83 mg∙kg^− 1^ (sample F7) to 622 mg∙kg^− 1^ (sample F8) (Table 3). The highest nickel contents were obtained for NPK-type compound fertilisers (samples F8 and F9). Taking into account that the permissible nickel content in inorganic macronutrient fertilisers is 100 mg∙kg^− 1^, it can be concluded that an elevated nickel content was found in the aforementioned samples. Particularly high nickel contents were found in fertiliser samples F3 and F4, i.e. in triple superphosphate.

The chromium content of the fertiliser samples ranged from 37.29 (sample F1) to 238 mg∙kg^− 1^ (sample F4), while its average content for all fertilisers tested was equal to 154 ± 30 mg∙kg^− 1^ (Table 3). The specified chromium content applies to both its trivalent form, which is insoluble and has low mobility, and the hexavalent form, which, due to its solubility and reactivity, may pose a serious health risk.

The lead concentration in the fertiliser samples ranged from 5.0 to 8.84 mg∙kg^− 1^ while the average lead content for all fertilisers tested was 5.43 ± 0.43 mg∙kg^− 1^. The lead content of phosphate fertilisers in the European Union ranges from 1.0 to 300 mg∙kg^− 1^, with an average of 26 mg∙kg^− 1^^45^. The limit value of 120 mg∙kg^− 1^ was not exceeded for any of the fertiliser samples tested. On this basis, it may be concluded that phosphate fertilisers produced in Poland contain significantly less lead in relation to the average concentration determined in the EU and in national regulations.

The cadmium content in the fertiliser samples ranged from 2.0 to 18.42 mg∙kg^− 1^ while its average content for all tested fertilisers was 7.87 ± 1.95 mg∙kg^− 1^. The cadmium content of phosphate fertilisers in the European Union is in the range 0.10–170 mg∙kg^− 1,^ with an average of 13 mg∙kg^− 1^ for this component^45^. On this basis, it may be concluded that phosphate fertilisers produced in Poland contain significantly less cadmium in their composition in relation to the average determined for fertilisers originating from the EU. The highest cadmium contents were found in samples F3 and F4, i.e. in triple superphosphate, which is also confirmed by other authors in their works^9,42^. According to literature data, cadmium content in Polish fertilisers ranges from 2.0 to 44.2 mg∙kg^− 1^^46^. The analysis of the fertiliser samples revealed that none of them exceeded the permissible cadmium content of 60 mg·kg⁻¹.

The zinc content of the fertiliser samples tested ranged from 70 to 410 mg∙kg^− 1^. The content of zinc in phosphate fertilisers in the European Union countries is within the range of 50–1450 mg∙kg^− 1^ and the average amounts 236 mg∙kg^− 1^^45^. On this basis, it may be concluded that phosphate fertilisers produced in Poland contain significantly less zinc in their composition in relation to the average determined for fertilisers originating from the EU countries. The highest zinc contents were found in samples F4 and F9, i.e. in triple superphosphate and NPK - type compound fertilizer.

The copper concentration in the fertiliser samples tested was in the range 10–66 mg∙kg^− 1^, while the average copper content for all fertilisers tested was 26 ± 7 mg∙kg^− 1^. The copper content of phosphate fertilisers used in the European Union is in the range 7.0–225 mg∙kg^− 1^, with an average of 13 mg∙kg^− 1^^45^. On this basis, it can be concluded that phosphate fertilisers produced in Poland contain more copper in their composition compared to the average for fertilisers originating from other EU countries. The highest copper contents were found in samples F3, F4 and F5, i.e. in triple superphosphate and NPK-type compound fertiliser.

In summary, it can be concluded that the tested fertilisers contain low amounts of heavy metals except samples F8 and F9, in which elevated nickel content was detected.

### The activity concentration of natural radionuclides

The results of the concentrations of natural radioactive isotopes in the tested fertiliser samples fluctuate over wide ranges (Table 3). A thorough analysis of the results reveals the presence of uranium isotope ^238^U in all of the fertilisers that were analysed. The concentration of this isotope ranged from 164 Bq∙kg^− 1^ for fertiliser sample F7 to 2140 Bq∙kg^− 1^ for fertiliser sample F4, with an average concentration of 715 Bq∙kg^− 1^. Given that the global average concentration of uranium in the Earth’s crust is 33 Bq∙kg^− 1 47^, it can be concluded that elevated concentrations of this isotope were obtained in all samples tested. In the case of sample F4, it was almost 65 times higher than the average concentration of ^238^U in the Earth’s crust.

**Table 3:** Activity concentrations of natural radionuclides ^238^U, ^226^Ra, ^232^Th and ^40^K in fertiliser samples.

Sample code	Activity concentrations (Bq∙ kg^− 1^)			
	^238^U	^226^Ra	^232^Th	^40^K
F1	254 ± 16	295 ± 18	40 ± 5	69 ± 5
F2	643 ± 45	665 ± 41	23 ± 2	278 ± 13
F3	1990 ± 127	1321 ± 80	14 ± 1	72 ± 5
F4	2140 ± 136	1492 ± 91	10.5 ± 0.9	60 ± 5
F5	212 ± 14	245 ± 15	15 ± 1	4399 ± 185
F6	314 ± 22	333 ± 21	14 ± 1	3432 ± 145
F7	164 ± 10	270 ± 17	14 ± 1	4980 ± 210
F8	342 ± 21	154 ± 10	4.2 ± 0.4	4908 ± 206
F9	376 ± 27	167 ± 11	3.8 ± 0.4	4050 ± 171
Average	715 ± 259	549 ± 170	12 ± 2	2472 ± 759
MIN	164	154	3.8	60
MAX	2140	1492	40	4980

The radium ^226^Ra isotope concentrations of the tested fertiliser samples ranged from 154 to 1492 Bq∙kg^− 1^, while its average concentration in the tested samples was equal to 549 Bq∙kg^− 1^. The lowest radium concentration, 154 Bq∙kg^− 1^, was measured for sample F8 of NPK compound fertiliser and the highest for sample F4 of TSP phosphate fertiliser (it was 1492 Bq∙kg^− 1^), similarly to the uranium isotope. Given that the average radium isotope concentration contained in the Earth’s crust is 32 Bq∙kg^− 1 47^. It can be concluded that, relative to this value, all fertilisers tested have several to even tens of times elevated concentrations of this isotope. In the case of fertiliser sample F4 (TSP), an increase of more than 46 times the radium isotope concentration was found in relation to its average concentration in the Earth’s crust.

The range of thorium isotope concentrations in the tested fertiliser samples was from 3.8 Bq∙kg^− 1^ to 40 Bq∙kg^− 1^, and its average concentration in the tested materials was 12 Bq∙kg^− 1^. The lowest thorium concentration was obtained for the NPK fertiliser sample F9, i.e. 3.8 Bq∙kg^− 1^, and the highest for the SSP phosphate fertiliser sample F1 which amounted 40 Bq∙kg^− 1^. According to the data presented in the UNSCEAR Report^47^ the average thorium isotope concentration in the Earth’s crust is at 45 Bq∙kg^− 1^. This means that all tested fertiliser samples, contain lower thorium isotope concentrations in relation to the thorium isotope concentration in the earth’s crust.

On the other hand, the range of potassium isotope concentrations in the studied fertiliser samples was very wide, i.e. from 60 to 4980 Bq∙kg^− 1^ with the average 2472 Bq∙kg^− 1^. While the lowest potassium concentrations were obtained for phosphate fertiliser samples, i.e. SSP and TSP. On the other hand, the highest potassium isotope concentrations were obtained for samples of NPK-type compound fertilisers. For both SSP and TSP fertiliser samples, the average potassium isotope concentration was lower than its average concentration in the Earth’s crust, which is 412 Bq∙kg^− 1 47^. In all multicomponent fertiliser samples, elevated potassium isotope concentrations of several to a dozen (eightfold for sample F6 and twelvefold for sample F7) were found compared to its average crustal concentration.

Taking into account all the results obtained from measurements of natural radioactive isotope concentrations for the mineral fertilisers studied, it can be concluded that the main influence on their elevated radioactivity is exerted by ^40^K > ^238^U > ^226^Ra > ^232^Th, respectively. Particularly hazardous in terms of alpha radioactivity are TSP fertilisers (samples F3 and F4), which contain in their composition high concentrations of ^238^U and ^226^Ra isotopes. Whereas, NPK-type fertilisers are distinguished by particularly high concentrations of ^40^K isotope.

The results of similar studies from different countries worldwide showed that phosphate and multicomponent mineral fertilisers vary in concentrations of natural radionuclides depending on the type and place of extraction of raw materials used for their production (Table 4).

**Table 4:** Natural radionuclide content (Bq∙kg^− 1^) in different types of fertilisers listed in the literature.

Country	^238^U	^226^Ra	^232^Th	^40^K	Reference
SSP					
Lebanon	4.15		0.25	13.43	^11^
Algeria		132 ± 4	12 ± 3	526 ± 39	^12,13^
Egypt	720 ± 7	1121 ± 10			^14^
Germany	749.6 ± 14.7	747.6 ± 7.3	-	-	^15^
Italy	670	295		-	^31^
Saudi Arabia	585 ± 35	392 ± 18		5029 ± 150	^32^
South Africa	85–98	55–90	316–327	-	^16^
Tanzania	3596–3879		408–434		^17^
Egypt (El-Mynia)		190.7	68.3	279.8	^18^
India	396.3 ± 9.7		39.3 ± 15.6	56.3 ± 34.3	^19^
USA	740	780			^23^
Germany	520	520			^23^
Poland	783–880	759–1074			^30^
Pakistan		526	50	221	^29^
Finland		54	11	3200	^21^
India		527 ± 15	7 ± 0.2	87 ± 2.0	^28^
TSP					
Algeria		156 ± 7	16 ± 2	534 ± 43	^12,13^
Germany	993.2 ± 8.7	250.1 ± 12.7	20.6 ± 1.8		^15^
Saudi Arabia	3900 ± 195	< 2.70		67.1 ± 8.50	^32^
India	284.2 ± 7.6		23.3	72.1 ± 49.9	^19^
USA (Florida)	2100	780		49	^23^
USA (Western)	1600	520		170	^23^
German	800	230		44	^23^
Poland	1790 ± 110	776 ± 9			^30^
Pakistan		558,6	84,8	142,5	^29^
Indonesia	558.66 ± 5.64	69.77 ± 0.94	4.09 ± 0.72	82.92 ± 3.27	^27^
Morocco	2524.42 ± 104.51	694.37 ± 201.58	3.97 ± 2.00	22.68 ± 5.55	^26^
NPK					
Lebanon	1.32		0.3	188.16	^11^
Algeria		149 ± 6	14 ± 3	3782 ± 250	^12,13^
Saudi Arabia	794 ± 44	35.8 ± 2.50		5709 ± 170	^32^
Germany	1062 ± 50		< 2.80	1760 ± 74	^32^
Poland	1140 ± 38	78 ± 2			^30^
India		79 ± 8	28 ± 0.6	1024 ± 11.7	^28^
Indonesia	311.94 ± 2.50	69.77 ± 1.23	21.60 ± 1.85	2925.05 ± 84.25	^27^
Morocco	825.77 ± 59.80	< 16.4	0.68 ± 0.68	4710.59 ± 236.70	^26^
USA		7.45 ± 2.76	2.63 ± 0.29	3581 ± 601	^25^
Iraq	143.154 ± 3.836		16.223 ± 0.650	93.458 ± 3.550	^24^
Pakistan	386		38	885	^20^
Finland		54	11	3200	^21^
Poland		4.91–173	4.68–27.9	2360–6110	^30^

The average concentrations of ^238^U, ^226^Ra, ^232^Th and ^40^K for the two SSP fertiliser samples (F1 and F2) from Poland amounted 449 ± 195, 480 ± 185, 18 ± 5 and 173 ± 104 Bq∙kg^− 1^, respectively. Similar uranium concentrations were obtained for SSP fertiliser samples originating from Italy, Saudi Arabia, India and Germany. Significantly higher uranium concentrations were determined in SSP fertilisers from Tanzania. In contrast, fertiliser samples from Lebanon and South Africa contained significantly lower concentrations of 238U compared to SSP fertiliser samples from Poland. The average concentration of the ^226^Ra isotope in the Polish SSP fertiliser samples is comparable to the average concentration of this isotope for fertiliser samples from Saudi Arabia, Germany, Pakistan and India. Much lower radium isotope concentrations were determined in fertiliser samples of the SSP type from Algeria, South Africa, Egypt (EL-Mynia) and Finland in relation to fertiliser samples of this type from Poland. Higher radium isotope concentrations than in tested fertilisers were obtained for samples originating from Egypt. Studies on Polish fertilisers of the SSP type were conducted by Olszewska-Wasiolek^30^and the results obtained by this author on the concentrations of both uranium and radium are higher concerning the fertiliser samples discussed in this paper. This is most likely since the phosphate rocks from which the SSP-type fertiliser was obtained contained higher concentrations of natural radioactive isotopes in their composition compared to the fertilisers investigated in the present work. Since the average concentration of the thorium isotope in the SSP fertiliser samples from Poland was 18 ± 5 Bq∙kg^− 1^, it is comparable to the concentration of this isotope for fertiliser samples from Algeria and Finland. Higher concentrations of ^232^Th were obtained for fertiliser samples from South Africa, Tanzania, Egypt (EL-Mynia) and Pakistan. In contrast, much lower concentrations of this isotope were obtained for fertilisers obtained from Lebanon and India.

For F3 and F4 TSP fertiliser samples, the average concentrations of uranium, radium, thorium and potassium were 2065 ± 75 Bq∙kg^− 1^, 1407 ± 86 Bq∙kg^− 1,^, 12 ± 2 Bq∙kg^− 1^and 66 ± 6 Bq∙kg^− 1^, respectively. Similar uranium concentrations were obtained for TSP fertiliser samples from the USA (Florida), USA (Western) and Poland. Significantly higher uranium concentrations were obtained for the materials tested in TSP fertilisers from Saudi Arabia. In contrast, fertiliser samples from Germany, India and Indonesia contained much lower concentrations of ^238^U than SSP fertiliser samples from Poland. The average concentrations of the ^226^Ra isotope in the TSP fertiliser samples studied are significantly higher (several or even more than a dozen times) compared to the results obtained by other researchers, both from Poland and abroad. Analysing the average thorium isotope concentration of 12 ± 2 Bq∙kg^− 1^ for the mentioned fertiliser samples with the results of different studies^11–32^it can be concluded that it is comparable to the average concentration of this isotope for fertiliser samples from Algeria. Higher concentrations of ^232^Th were obtained for fertiliser samples from Germany, India and Pakistan. In contrast, lower concentrations of this isotope were obtained for fertilisers originating from Indonesia and Morocco. The average concentration of the ^40^K isotope in the F3 and F4 fertiliser samples was 66 ± 6 Bq∙kg^− 1,^ which is comparable to its average concentration for the TSP fertiliser samples from Saudi Arabia and India. Higher ^40^K isotope concentrations for the materials tested for TSP-type fertilisers from Algeria, the USA (Western), Pakistan and Indonesia were obtained. In contrast, fertiliser samples from the USA (Florida), Germany and Morocco contained lower potassium isotope concentrations than samples of this type of fertiliser from Poland.

For NPK fertilisers from Poland (samples F5 to F9), the average concentrations of ^238^U, ^226^Ra, ^232^Th and ^40^K were 282 ± 40 Bq∙kg^− 1^, 234 ± 33 Bq∙kg^− 1^, 10 ± 3 Bq∙kg^− 1^and 4354 ± 287 Bq∙kg^− 1^, respectively. Similar uranium concentrations were obtained for NPK fertiliser samples from Indonesia and Pakistan. While significantly higher uranium concentrations for the materials tested were obtained for fertilisers from Saudi Arabia, Germany, Poland and Morocco. In contrast, NPK fertiliser samples from Lebanon and Iraq contained much lower concentrations of ^238^U than samples of this fertiliser from Poland. The average radium isotope concentration in the samples tested was higher than for fertilisers from countries around the World, in that from Poland. This is most likely due to contamination of the raw materials (i.e. phosphate rock) from which the tested fertilisers were obtained. Taking into account the fact that Poland does not have phosphate rock deposits, it is forced to import them from other countries, i.e. from Morocco, Algeria, Tunisia, the USA and China. However, the concentration of natural radioactive isotopes in phosphorites is not uniform and depends on where they are mined, e.g. the average concentration of radium and uranium in phosphorites from Morocco is 1600 Bq∙kg^− 1^ (^226^Ra) and 1700 Bq∙kg^− 1^ (^238^U), respectively, and in fertilisers from Syria is 300 Bq∙kg^− 1^ (^226^Ra) and 1000 Bq∙kg^− 1^ (^(238)^U), respectively^48^. Then, the average thorium isotope concentration in the studied samples of NPK-type fertilisers from Poland is comparable to the average concentration of this isotope for fertiliser samples from Algeria and Finland. Lower thorium isotope concentrations, in relation to fertilisers from Poland, were obtained for samples of this type of fertilisers from Lebanon, Germany, Morocco and the USA. While higher thorium isotope concentrations in relation to the fertilisers studied were obtained by researchers for samples originating from India, Iraq and Pakistan. The compare of the average potassium isotope concentration of NPK fertiliser samples with the results of other researchers, showed that it is comparable to that of fertiliser samples from Algeria Morocco and the USA. In contrast, higher concentrations of ^40^K were obtained for fertiliser samples from Saudi Arabia and lower concentrations for fertilisers from Lebanon, Germany, India, Iraq, Pakistan, Finland and Indonesia.

### The correlation analysis

A correlation analysis was performed in order to ascertain the relationships between the individual components of the tested fertilisers. This analysis yielded only a few significant relationships, i.e. those for which the confidence level p was below 0.05 (Table 5). In the majority of cases, the confidence level is much higher, so despite the high correlation coefficient, these values cannot be considered statistically significant. In this context, it can be concluded that uranium, nickel, zinc, and copper did not demonstrate a clear relationship with any of the heavy metals, macronutrients, or natural radioactive isotopes. The calculated correlation coefficients indicate that the most significant associations are observed for ^226^Ra, which correlates with ^40^K (*r* = -.7345 and *p* = .024), Cr (*r* = .7910 for *p* = .011), Cd (*r* = .9753, *p* = .000), and P (*r* = .9706 and *p* = .000). A significant correlation exists between chromium and not only ^226^Ra, but also Cd and P. The correlation coefficients between these parameters were determined to be 0.7863 for *p* = .012 and 0.7911 for *p* = .011, respectively. A clear and significant correlation was also found between Pb and ^232^Th (*r* = .8452 for *p* = .004) and a very strong one between Cd and P (*r* = .9076, *p* = .001). The significant correlations found between ^226^Ra and ^40^K, as well as between P and ^40^K, are negative, i.e. an increase in the ^40^K concentration is associated with a decrease in the concentrations of both ^226^Ra and P. It is generally assumed that high and significant correlation coefficients indicate a common source for the studied elements. The results presented herein indicate that ^226^Ra, K, Cr, Cd, and P are likely to have a common source, which is most likely phosphate rocks.

**Table 5:** Correlation coefficients (r) between macronutrient content of heavy metals and natural radioactive isotopes in fertiliser samples tested.

Variables	^238^U	^226^Ra	^232^Th	^40^K	Ni	Cr	Pb	Cd	*P*	Zn	Cu
^238^U	1.0000										
	p= ---										
^226^Ra	0.5525	1.0000									
	*p* = .123	p= ---									
^232^Th	− 0.0031	− 0.0249	1.0000								
	*p* = .994	*p* = .949	p= ---								
^40^K	− 0.4922	**− 0.7345**	− 0.5645	1.0000							
	*p* = .178	*p* = .024	*p* = .113	p= ---							
Ni	− 0.0758	− 0.3329	− 0.4808	0.4380	1.0000						
	*p* = .846	*p* = .381	*p* = .190	*p* = .238	p= ---						
Cr	0.4181	**0.7910**	− 0.4435	− 0.3566	0.2259	1.0000					
	*p* = .263	*p* = .011	*p* = .232	*p* = .346	*p* = .559	p= ---					
Pb	− 0.2221	− 0.1868	**0.8452**	− 0.3956	− 0.1531	− 0.3094	1.0000				
	*p* = .566	*p* = .630	*p* = .004	*p* = .292	*p* = .694	*p* = .418	p= ---				
Cd	0.5511	**0.9753**	− 0.1829	− 0.6198	− 0.3209	**0.7863**	− 0.3757	1.0000			
	*p* = .124	*p* = .000	*p* = .638	*p* = .075	*p* = .400	*p* = .012	*p* = .319	p= ---			
P	0.4357	**0.9706**	0.0678	**− 0.7957**	− 0.2720	**0.7911**	− 0.0155	**0.9076**	1.0000		
	*p* = .241	*p* = .000	*p* = .862	*p* = .010	*p* = .479	*p* = .011	*p* = .968	*p* = .001	p= ---		
Zn	0.2667	0.5539	− 0.4621	− 0.3158	− 0.0923	0.6368	− 0.3312	0.6047	0.5478	1.0000	
	*p* = .488	*p* = .122	*p* = .210	*p* = .408	*p* = .813	*p* = .065	*p* = .384	*p* = .085	*p* = .127	p= ---	
Cu	0.0336	0.4452	− 0.1152	− 0.1446	− 0.2214	0.3464	− 0.2261	0.4683	0.4227	0.0731	1.0000
	*p* = .932	*p* = .230	*p* = .768	*p* = .710	*p* = .567	*p* = .361	*p* = .559	*p* = .204	*p* = .257	*p* = .852	p= ---

### The radiological hazard assessment or the radiological hazard indicators

**Fig. 2:**
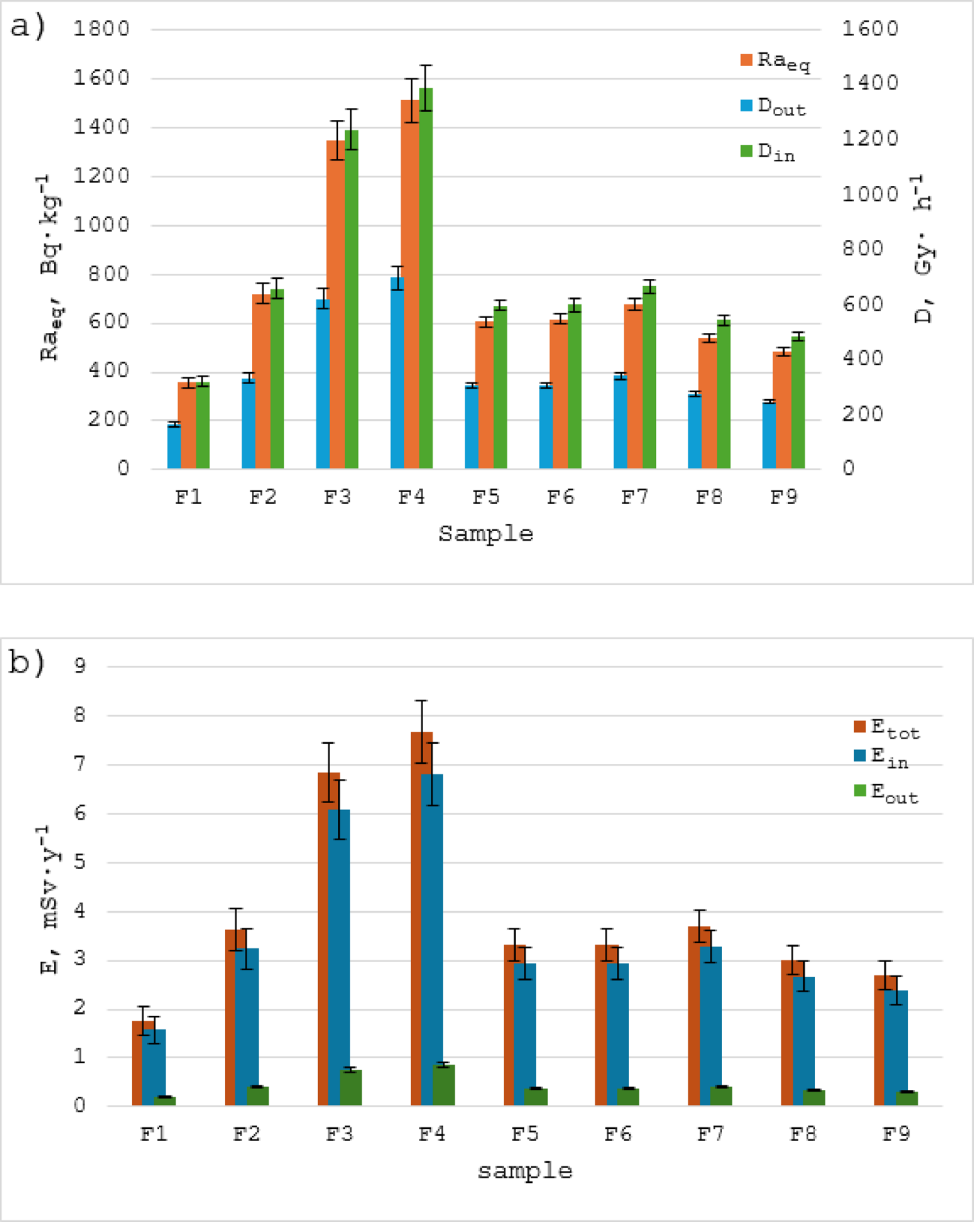
Values of radiation hazard indices calculated for samples of mineral/synthetic fertilisers: (**a**) radium equivalent activity (Ra_eq_), outdoor dose rate (D_out_), indoor dose rate (D_in)_; (**b**) annual outdoor effective dose(E_out_ ), annual indoor effective dose (E_in_), total annual effective dose (E_tot_).

To assess the exposure of people to ionising radiation emitted by phosphate and compound fertilisers, the basic radiation hazard parameters have been determined, i.e. radium equivalent activity (Ra_eq_), outdoor dose rate (D_out_), indoor dose rate (D_in)_, annual outdoor effective dose and annual indoor effective dose (E_out_ and E_in_) as well as total annual effective dose (E_tot_) ( Fig. 2; Table 6). The values of these components are especially important for the exposure assessment of workers who have direct contact with fertilisers, i.e. fertiliser factory workers, people working in the loading, packaging, transport and sale of fertilisers and also direct users, i.e. farmers.

**Table 6:** Values of radiological hazard indicators: radium equivalent activity (Ra_eq_), outdoor dose rate (D_out_), indoor dose rate (D_in_), annual outdoor effective dose and annual indoor effective dose (E_out_ and E_in_) as well as total annual effective dose (E_tot_) in the studied types of fertiliser samples

Sample code	Ra_eq_ (Bq∙kg^− 1^)	D_out_ (nGy∙ ^h(−1)^)	D_in_ (nGy∙ ^h(−1)^)	E_out_ (mSv∙y^− 1^)	E_in_ (mSv∙y^− 1^)	E_tot_ (mSv∙y^− 1^)
MIN	357	163	321	0.20	1.57	1.77
MAX	1512	698	1386	0.86	6.82	7.68
Average	761 ± 132	366 ± 59	722 ± 118	0.45 ± 0.07	3.55 ± 0.58	3.99 ± 0.65
World average	≤ 370	58	84	0.07	0.41	0.48

The radium equivalent activity (Ra_eq_) is the weighted sum of activities of ^226^Ra, ^232^Th, and ^40^K in a fertiliser. It is based on the assumption that 370 Bq∙kg^− 1^ of ^226^Ra, 259 Bq∙kg^− 1^ of ^232^Th, and 4810 Bq∙kg^− 1^ of ^40^K produce the same gamma-radiation dose rate^49^. The values of the (Ra_eq_) calculated for the mineral fertilisers range from 357 to 1512 Bq∙kg^− 1^ with an average of 761 ± 132 Bq∙kg^− 1^. The average is more than twice as high as the world’s average, which amounts 370 Bq∙kg^− 1 47^. The highest Ra_eq_ values were obtained for triple superphosphate fertiliser samples (F3 and F4).

The outdoor doses rate (D_out_) due to the presence of ^226^Ra, ^232^Th and ^40^K in the tested samples of fertilisers were calculated and their values vary from 163 to 698 nGy∙kg^− 1^, with an average value of 366 ± 59 nGy∙kg^− 1^ (Table 6). It turns out that this value is about 6.3 times higher than the global average, which is equal to 58 nGy∙kg^− 1 47^. The highest value D_out_ was obtained for the triple superphosphate fertiliser samples (F3 and F4).

The values of indoor dose rate (D_in_) are calculated during this study range from 321 to 1386 nGy∙kg^− 1^, with an average of 722 ± 118 nGy∙kg^− 1^, which is 8.6 times higher than the world’s average of 84 nGy∙kg^− 1 47^. The highest value of D_out_ was obtained for triple superphosphate fertiliser samples (F3 and F4) as in the case of Ra_eq_ and D_out_.

The value of annual outdoor effective dose (E_out_) ranges from 0.20 to 0.86 mSv∙y^− 1^, with an average of 0.45 ± 0.07 mSv∙y^− 1^, which is 6.4 times higher than its world’s average of 0.07 mSv∙y^− 1 49^. in this case, the highest E_out_ value was also obtained for the triple superphosphate fertiliser samples (F3 and F4).

The values of annual indoor effective dose (E_in)_ calculated for the tested samples of mineral fertilisers are presented in Table 6. It ranges from 1.57 to 6.82 mSv∙y^− 1^ with an average of 3.55 ± 0.58 mSv∙y^− 1^, which is 8.6 times higher than the world’s average of 0.41 mSv∙y^− 1 47^. The total annual effective dose (E_tot_) was estimated at 3.99 ± 0.65 mSv∙y^− 1^, which is 8.3 times higher than the world’s average (0.48 mSv∙y^− 1^) and also much higher than the criterion limit of 1 mSv∙y^− 1^ as per ICRP^50^.

A thorough analysis of the obtained results reveals that all the fertilisers that were examined were found to have elevated radiological indicators. This suggests a potential health risk to individuals who have regular and prolonged contact with these fertilisers. Production workers involved in the handling, packing and transport of fertilisers, as well as farmers who utilise these fertilisers on their agricultural land, are particularly vulnerable. The bioavailable doses of radionuclides are received by humans through multiple pathways, including ingestion, inhalation (for radon and airborne particles), absorption, and injection. It is widely acknowledged that radionuclides are prone to undergoing a multitude of transformations and reactions within the human body, with the potential to induce a plethora of adverse effects. These include damage to chromosome material, cell apoptosis, and mutation. It is particularly dangerous when exposure is prolonged, as it can cause epilation, chronic lung diseases, acute leukopenia, anaemia, skin burns, and necrosis of the mouth^51^. It is therefore important that production workers and farmers use personal protective equipment when working with this type of fertiliser, in order to avoid exposure to additional doses of ionising radiation. A further potential threat is the long-term environmental impact caused by elevated levels of radionuclides in the soil resulting from repeated fertiliser applications. This leads to the accumulation of radionuclides in the soil, as well as to runoff into groundwater and surface water, and uptake by plants.

## Conclusion

In the paper, the activity concentrations of the natural radionuclides ^238^U, ^226^Ra, ^232^Th and ^40^K in 9 fertilisers samples were determined. The average concentrations of ^238^U, ^226^Ra, ^232^Th and ^40^K were equal to 715 Bq∙kg^− 1^, 549 Bq∙kg^− 1^, 12 Bq∙kg^− 1^ and 2472 Bq∙kg^− 1^, respectively. Considering that the average concentrations of ^238^U, ^(226^Ra), ^232^Th and ^40^K in the Earth’s crust are respectively: 33, 32, 45 and 412 Bq∙kg^− 1^, it can be concluded that fertilisation of the soils with the phosphate and compound fertilisers studied will enrich the soil with natural radioactive isotopes.

Although the fertilisers tested contain heavy metals such as nickel, chromium, lead, cadmium, zinc and copper in their composition, their concentrations are so low that they should not significantly affect soil contamination. The exceptions are two samples of triple superphosphate fertilisers (F3 and F4), in which significantly elevated nickel contents were detected. The calculated correlation coefficients between individual heavy metals, macronutrients and natural radioactive isotopes in the analysed fertilisers indicate that there are statistically significant high correlations between them, such as in the case of Cd and ^226^Ra (*r* = .9753), P and ^226^Ra ( *r* = .9706) and between Cd and P ( *r* = .9074).

The results obtained for the concentrations of natural radioactive isotopes allowed to determination the most important radiological protection parameters, such as radium equivalent activity (Ra_eq_), outdoor dose rate (D_out)_ indoor dose rate (D_in)_, as well as annual outdoor effective dose and annual indoor effective dose (E_out_ and E_in_) and total annual effective dose (E_tot_). The average values of these parameters obtained for the fertilisers tested were much higher than their global average values. Special attention should be paid to the total effective dose, which was higher than 1 mSv∙y^− 1^ for all tested fertiliser samples. Particularly high effective doses were obtained for superphosphate triple fertiliser samples F3 and F4, which amounted 6.83 and 7.68 mSv∙y^− 1^, respectively. Such high values for this parameter indicate a potentially high health risk caused by the use of these fertilisers. Therefore, it is recommended that workers who have direct contact with the tested fertilisers, i.e. primarily factory workers, loading and unloading personnel, transporters, warehousemen and farmers, take appropriate precautions (i.e. use of masks, gloves and minimising the time spent working with fertilisers) so that the doses they receive are as low as possible.

## Supplementary Information

Below is the link to the electronic supplementary material.

Supplementary Material 1

## Data Availability

The data presented in this study are available, as they are included in the supplementary material.
